# Parabrachial nucleus astrocytes regulate wakefulness and isoflurane anesthesia in mice

**DOI:** 10.3389/fphar.2022.991238

**Published:** 2023-01-13

**Authors:** Pei-Chang Liu, Wei Yao, Xing-Yu Chen, Wei-Kun Su, Ze-Hong Zheng, Xiong-Bin Yan, Ya-Ling Deng, Kai-Ge Shi, Xin Liu, Yu-Wei Gao, Tian-Tian Lin, Yun-Xi Zhu, Ying-Xuan Lin, Zhong-Hua Zhu, Ping Cai, Liang-Cheng Zhang, Li Chen

**Affiliations:** ^1^ Department of Anesthesiology, Fujian Medical University Union Hospital, Fuzhou, Fujian, China; ^2^ Fujian Province Key Laboratory of Environment and Health, School of Public Health, Fujian Medical University, Fuzhou, Fujian, China; ^3^ Department of Pharmacology, School of Pharmacy, Fujian Medical University, Fuzhou, Fujian, China; ^4^ School of Basic Medical Sciences, Fujian Medical University, Fuzhou, Fujian, China

**Keywords:** parabrachial nuclei, astrocytes, sleep-wake, general anesthesia, isoflurane

## Abstract

**Background:** The parabrachial nucleus (PBN) is an important structure regulating the sleep–wake behavior and general anesthesia. Astrocytes in the central nervous system modulate neuronal activity and consequential behavior. However, the specific role of the parabrachial nucleus astrocytes in regulating the sleep-wake behavior and general anesthesia remains unclear.

**Methods:** We used chemogenetic approach to activate or inhibit the activity of PBN astrocytes by injecting AAV-GFAabc1d-hM3Dq-eGFP or AAV-GFAabc1d-hM4Di-eGFP into the PBN. We investigated the effects of intraperitoneal injection of CNO or vehicle on the amount of wakefulness, NREM sleep and REM sleep in sleep–wake behavior, and on the time of loss of righting reflex, time of recovery of righting reflex, sensitivity to isoflurane, electroencephalogram (EEG) power spectrum and burst suppression ratio (BSR) in isoflurane anesthesia.

**Results:** The activation of PBN astrocytes increased wakefulness amount for 4 h, while the inhibition of PBN astrocytes decreased total amount of wakefulness during the 3-hour post-injection period. Chemogenetic activation of PBN astrocytes decreased isoflurane sensitivity and shortened the emergence time from isoflurane-induced general anesthesia. Cortical EEG recordings revealed that PBN astrocyte activation decreased the EEG delta power and BSR during isoflurane anesthesia. Chemogenetic Inhibition of PBN astrocytes increased the EEG delta power and BSR during isoflurane anesthesia.

**Conclusion:** PBN astrocytes are a key neural substrate regulating wakefulness and emergence from isoflurane anesthesia.

## 1 Introduction

Inhaled anesthetics enable patients to undergo surgery without consciousness and pain, benefiting millions of surgical patients annually. Inhaled anesthetics have been used clinically for over 170 years, yet the underlying mechanisms remain unclear. Recently, wakefulness or sleep-promoting nuclei have been shown to be involved in the induction and, maintenance, and emergence from anesthesia (Wang et al., 2019; Reitz et al., 2020; Bao et al., 2021), suggesting that general anesthesia may share some neural circuits with sleep-wake behavior (Yang et al., 2021).

The parabrachial nucleus (PBN), an important component of the pons, is a key node of the ascending reticular activating system (Fuller et al., 2011). Previous studies have shown that PBN neurons, especially glutamatergic neurons, play an important role in regulating sleep -wake behavior and general anesthesia. Chemogenetic activation of PBN neurons induced approximately 11 h of sustained arousal in mice without sleep rebound (Qiu et al., 2016). Our previous study showed that glutamatergic neurons in the medial PBN are essential in controlling wakefulness and, drive cortical arousal and behavioral wakefulness *via* the basal forebrain (BF) and lateral hypothalamus (LH) (Xu et al., 2021). PBN neuron activation promotes cortical arousal and behavioral emergence from isoflurane and propofol anesthesia in rats (Luo et al., 2018). Selective activation of glutamatergic neurons in the PBN accelerates reanimation from inhalational general anesthesia in mice (Wang et al., 2019).

Recently, numerous studies have shown that central nervous system astrocytes influence neuronal activity and regulate sleep-wake behavior and general anesthesia. By *in vivo* calcium recording, Ingiosi and colleagues demonstrated that the activity of cortical astrocytes synchronously changed with the sleep -wake transition (Ingiosi et al., 2020). Optogenetic stimulation of astrocytes in the ventrolateral preoptic area (VLPO) increased the extracellular ATP concentration and c-Fos expression in sleep-promoting VLPO neurons, and increased active phase sleep in rats (Kim et al., 2020). Mice with the mitochondrial astrocyte gene NDUFS4 specifically knocked out show a profound hypersensitivity to volatile anesthetics, revealing that the normal function of astrocytes is essential for emergence from general anesthesia (Ramadasan-Nair et al., 2019). However, the role of astrocytes in the PBN in sleep-wake behavior and general anesthesia remains unknown.

In the present study, we used chemogenetic approaches to manipulate the activity of PBN astrocytes and investigated their role in regulating sleep -wake behavior and isoflurane -induced general anesthesia. Our results showed that PBN astrocyte activation induced an increase in wakefulness, whereas inhibition reduced total wakefulness. Chemogentic activation of PBN astrocytes decreased isoflurane sensitivity and accelerated emergence from isoflurane anesthesia. Electroencephalogram (EEG) spectral analyses and burst suppression ratio (BSR) calculations indicated the enhancement of cortical activity after PBN astrocyte activation and attenuation of cortical activity after PBN astrocyte inhibition. Collectively, our results uncover an important role of PBN astrocytes in regulating sleep -wake behavior and isoflurane anesthesia.

## 2 Materials and methods

### 2.1 Animals

The C57BL/6 mice (aged 8–10 weeks and weighing 25–30 g) used in this experiment were purchased from Shanghai SLAC Laboratory Animal. The animals were housed in an environment with an independent ventilation system at constant temperature (22°C ± .5°C) and relative humidity (60 ± 2%) with a 12-h light/12-h dark cycle. The mice had free access to food and water. All the experimental procedures of this project were conducted in accordance with the guidlines of the Ethics Committee of Fujian Medical University.

### 2.2 Virus injection

Mice were anesthetized with 3% isoflurane and placed on a stereotactic device (RWD Life Science, China). The skin was incised along the centerline, and a small craniotomy was made above the PBN after positioning according to the bregma. AAV-GFAabc1d-hM3Dq-eGFP (S0482, Taitool, China), AAV-GFAabc1d-hM4Di-eGFP (S0489, Taitool, China), or AAV-GFAabc1d-eGFP (S0246, Taitool, China) were bilaterally microinjected into the PBN (Coordinates: AP: 5.20; ML: +1.20; DV: 3.25). The glass pipette was left at the injection site for an additional 10 min to allow the virus to diffuse, then moved up .1 mm and slowly removed after 5 min. After the virus injection was completed, the EEG electrodes were screwed into the craniotomy holes, and two Teflon-coated stainless-steel wires were bilaterally placed into the trapezius muscles and used as electromyogram (EMG) electrodes. EEG and EMG electrodes were attached to a miniconnector which was fixed on the skull surface with dental cement.

### 2.3 Polysomnographic recording in sleep-wake behavior

Two weeks after viral injection and two weeks before the polysomnographic recording, the mice were housed separately in a transparent home cage and accommodated to a 12-h light/12-h dark cycle, in which the onset of the light period was defined as Zeitgeber Time 00:00 (ZT 00:00) and the onset of the dark period was defined as ZT 12:00. The sleep monitoring system (Biotex Kyoto, Japan) was used to record the EEG/EMG. After adaptation, the same mice were intraperitoneally injected with vehicle and clozapine-N-oxide (CNO, 1 mg/kg, A3317, APExBIO) successively. CNO injection was performed 24 h after vehicle injection. Sleep states were automatically scored using SleepSign software (Kissei Comtec, Japan) and corrected manually if necessary.

### 2.4 Assessment of sensitivity to isoflurane

Loss of righting reflex (LORR) and recovery of righting reflex (RORR) were assessed as previously described (Wang et al., 2019). LORR and RORR assessments were conducted in an acrylic anesthesia chamber connected to an isoflurane vaporizer and an infrared gas monitor on opposite sides of the box. The chamber received isoflurane (R510-22; RWD Life science; China) inflow in an air carrier (1.5 L/min) from an isoflurane vaporizer, and the infrared gas monitor monitored the isoflurane concentration. To determine the dose-response curve for LORR or RORR, mice were intraperitoneally injected with CNO (3 mg/kg) or vehicle 1 h before anesthesia induction. At the beginning of the dose-response experiment for LORR, isoflurane was administered to the chamber starting at a concentration of 0%, and the concentration increased in increments of .1% every 15 min until LORR occurred. In the dose-response experiment for RORR, isoflurane was initially administered at a concentration of 1.4% and then gradually decreased by .1% every 15 min until RORR occurred. After a 15 min equilibration period with anesthetic gas, the chamber was rotated to place the mouse on its back. LORR was defined if the mouse did not twist the body and place all four feet on the floor within 30 s in at two repeat operations, whereas RORR was defined when the mouse twisted from the supine position and placed all four feet on the floor within 30 s. In all experiments, the temperature of the mice was maintained by placing a 37°C heating pad beneath the chamber.

### 2.5 Assessment of induction and emergence times

An isoflurane concentration of 1.4% was used to assess isoflurane anesthesia induction and emergence times. Mice were intraperitoneally injected with CNO (3 mg/kg) or vehicle 1 h before anesthesia induction. The isoflurane concentration in the anesthesia induction box was monitored and maintained at 1.4% during the process. The mouse was placed into the anesthetic chamber, and the cage was rotated to place the mouse on its back every 10 s. The LORR was defined when the mouse did not twist the body and place all four feet on the floor within 30 s. After a 30 min exposure to 1.4% isoflurane, the mouse was removed from the anesthetic chamber, and placed in a supine position in room air. Emergence time was defined as the duration from the time of removal from the isoflurane chamber to the time the mouse righted itself (all feet on the floor).

### 2.6 EEG recording and analysis in isoflurane anesthesia

The amplified EEG and EMG data were collected at a 128- Hz sampling rate. For EEG/EMG signals recording, mice were intraperitoneally injected with vehicle or 3 mg/kg CNO 30 min before the test. The EEG/EMG of the mice was monitored immediately after they were placed put into the anesthesia induction box. The mice were placed into an anesthesia induction chamber filled with .8% or 1.0% isoflurane for 30 min, then EEG signals were recorded for 30 min. We analyzed the EEG power spectrum in isoflurane experiments according to a method described before with some modifications (Bao et al., 2021). Briefly, the EEG power spectra were firstly segmented into consecutive .25 Hz bins within the frequency range of 0–30 Hz by SleepSign software (Kissei Comtec, Japan). Then, the EEG power spectrum was further standardized by expressing each band power as a percentage of the total power to obtain the relative power densities [for example, band power (.5–.75 Hz)/total power (0–30 Hz)]. To compare the difference of EEG power spectra between CNO and vehicle injection, the relative power densities of EEG signals (CNO, injection) is subtracted that of EEG signals (vehicle injection) (for example, band power (0.5Hz–.75 Hz)/total power (0–30 Hz) with CNO injection subtract band power (.5–.75 Hz)/total power (0–30 Hz) with vehicle injection). The relative power bands attributed to delta (.5–4 Hz), theta (4–7 Hz), alpha (8–15 Hz), and beta (16–30 Hz) was summed separately.

For BSR calculations, EEG data were scored and divided into burst and suppression portions by EEG amplitude, as previously described (Li et al., 2019; Yin et al., 2019). If the amplitude of the EEG was less than the interval threshold, the brain wave was classified as a suppressed waveform and assigned a value of 1. If the EEG amplitude was greater than the interval threshold, the brain wave was classified as a burst and assigned a value of 0. The BSR was calculated as the percentage of event 1 to event 0 and 1. The minimum duration of burst and suppression periods was .5 s.

### 2.7 Immunofluorescence staining

At the end of all experiments, mice were intraperitoneally injected with CNO or vehicle for 1 h, and then deeply anesthetized with sodium pentobarbital. After perfusion with .01 M phosphate buffer saline (PBS), the brain tissues were removed and post-fixed with 4% paraformaldehyde then placed in 20% and 30% sucrose solutions for dehydration at 4°C. The brain tissue was sliced and mounted on a slide. The slices were washed with PBS and incubated with .7% TritonX100 in a 37°C-incubator for 1 h. The slices were then incubated with mouse-anti-GFAP antibody (1:1,000, MA5-12023, Invitrogen) or mouse-anti-NeuN antibody (1:1,000, MAB377, Millipore) at 37°C for 24 h. Then, the slices were incubated with donkey-anti-mouse-Alexa Fluor 594 (1:1,000, 715–585-151, Jackson) at 37°C for 2 h after 15 min PBS washes.

### 2.8 Statistics

We used the sleep analysis system (SleepSign3-OBI) to analyze EEG and GraphPad8.0 to analyze statistical data. All data are expressed as the mean ± SEM. The statistical methods used include *t*-tests and two-way repeated-measure ANOVA. If there was a significant difference, multiple pairwise comparisons between groups were conducted. *p* < .05 was considered to represent significance. The description is as follows: *, *p* < .05; **, *p* < .01; ***, *p* < .001. Adobe Illustrator CS6 was used to lay out all the graphics.

## 3 Results

### 3.1 Chemogenetic activation of PBN astrocytes promotes wakefulness

**FIGURE 1:**
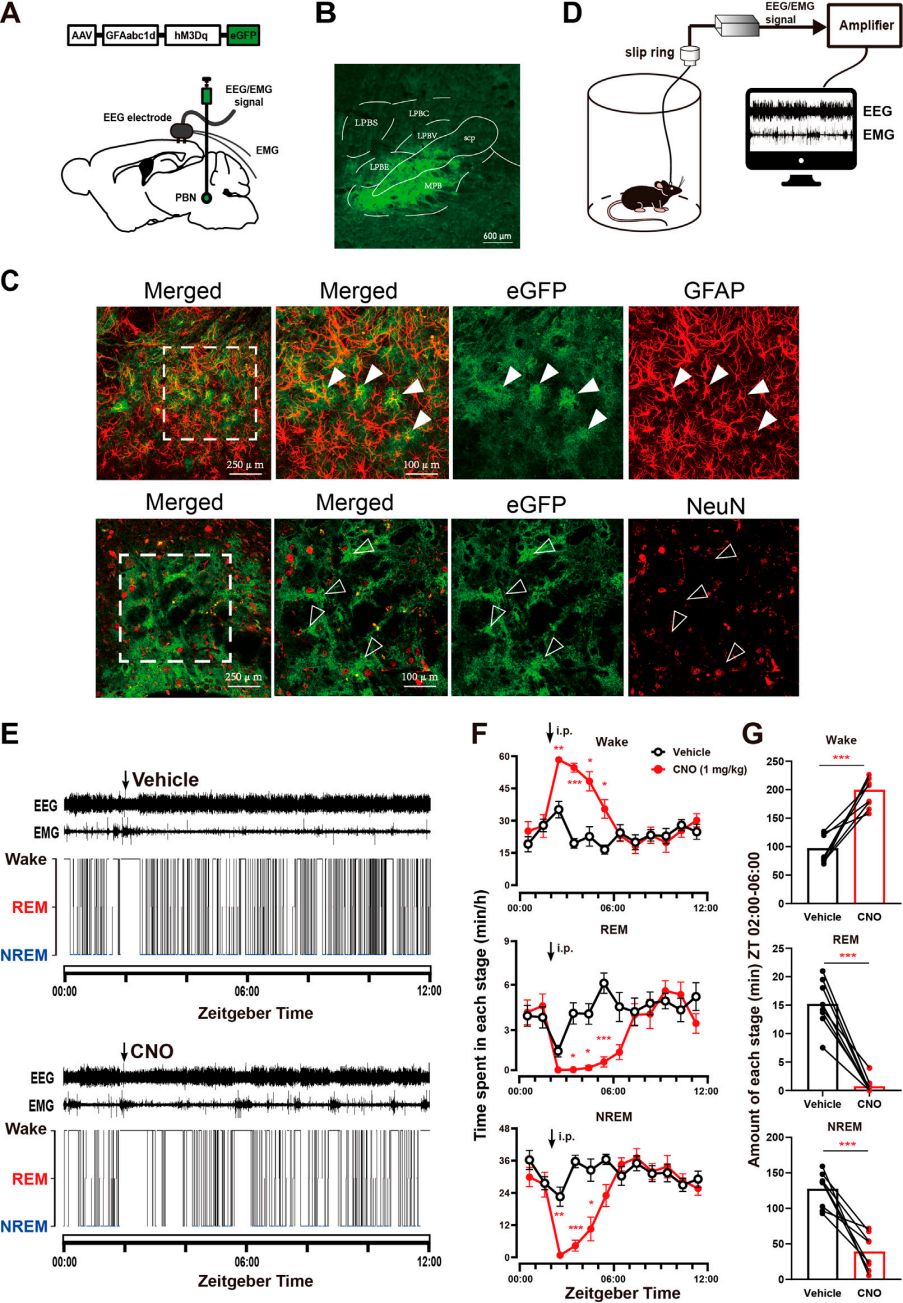
Chemogenetic activation of PBN astrocytes increases wakefulness **(A)** Schematic diagram showing the bilateral injection of AAV-GFAabc1d-hM3Dq-eGFP into the PBN of mice. The electroencephalogram (EEG) electrodes were screwed into the craniotomy hole, and the electromyogram (EMG) electrodes were bilaterally placed into the trapezius muscles. **(B)** Representative fluorescent image showing the expression of AAV-GFAabc1d-hM3Dq-eGFP in the PBN, scale bar = 600 μm. LPBC, the central part of the lateral parabrachial nucleus; LPBE, the external part of the lateral parabrachial nucleus; LPBS, the superior part of the lateral parabrachial nucleus; LPBV, the ventral part of the lateral parabrachial nucleus; MPB, medial parabrachial nucleus; scp, superior cerebellar peduncle. **(C)**Immunofluorescence showing the colocalization of GFAP and eGFP (top), and the colocalization of NeuN and eGFP (bottom), closed arrow in shows a GFAP-positive cell. Open arrow shows a NeuN-negative astrocyte. Scale bar = 250 μm or 100 μm. GFAP, glial fibrillary acidic protein; NeuN, neuronal nuclei. **(D)** Schematic of the configuration of the electroencephalographic (EEG)/electromyographic (EMG) with EEG/EMG recording system. **(E)** Typical examples of EEG/EMG traces and corresponding hypnograms from one hM3Dq mouse which was intraperitoneally injected with vehicle (top) or CNO (1 mg/kg, bottom) successively at ZT 02:00. CNO injection was performed 24 h after vehicle injection. **(F)** Time course of wakefulness, REM sleep, and NREM sleep after intraperitoneal injection of vehicle or 1 mg/kg CNO in hM3Dq mice. Two-way repeated-measures ANOVA (*n* = 8). **(G)** Total time spent in each stage during the 4-h post-injection period (ZT 02:00–06:00) in hM3Dq mice. Paired *t*-test (*n* = 8). Values represent the mean ± SEM; *p < .05, **p < .01 or ***p < .001, indicates significant differences between the vehicle and experimental groups. PBN, parabrachial nucleus; CNO, clozapine N-oxide; i.p., intraperitoneal injection.

To investigate whether PBN astrocytes participate in sleep-wake regulation, we first tested the effect of chemogenetic activation of PBN astrocytes. To selectively activate PBN astrocytes, we bilaterally microinjected an excitatory chemogenetic virus, AAV-GFAabc1d-hM3Dq-eGFP, into the PBN of mice (Figure 1A). The virus is under the control of the gfaABC1D promoter (Cui et al., 2018; Kim et al., 2020). After approximately four weeks, robust fluorescence of eGFP was observed in the PBN (Figure 1B and Supplementary Figure S1). To test the cell type specificity of the viral vectors, we performed immunofluorescence staining for GFAP (a marker for astrocytes) and NeuN (a marker for neurons). Our results showed that the eGFP-expressing cells were mostly GFAP -expressing, and NeuN rarely co-labeled eGFP-expressing cells (Figure 1C). The Donkey-anti-mouse-Alexa Fluor 594 (secondary antibodies) used alone did not cause false positive immunolabeling (Supplementary Figure S2). The cell type specificity of GFAabc1d-hM3Dq-eGFP was also demonstrated in our previous study (Cai et al., 2022). The EEG electrodes were screwed into the craniotomy hole and touched the cortex, and the EMG electrodes were bilaterally placed into the trapezius muscles, and the EEG/EMG signals were recorded to judge the sleep–wake states of the mice (Figure 1D). The vehicle injection produced a typical light phase hypnogram with dominant NREM sleep, marked by high EEG delta power and low EMG activity interrupted by bouts of wakefulness (Figure 1E). Our results show that 1 mg/kg CNO injection induced sustained wakefulness and produced more longer bouts of wakefulness. CNO injection resulted in a significant increase in wakefulness from ZT 02:00 to ZT 06:00, with a significant decrease in NREM and REM sleep, compared to the vehicle injection (Figure 1F). During the 4-h post-injection period (from ZT 02:00 to ZT 06:00), the total level of wakefulness increased significantly, while NREM sleep and REM sleep significantly decreased (Wake, 200.05 ± 9.52 min at CNO injection vs. 97.15 ± 9.09 min at vehicle injection, *n* = 8, *p* = .0003; NREM, 39.21 ± 9.20 min at CNO injection vs. 127.60 ± 9.05 min at vehicle injection, *n* = 8, *p* = .0006; REM, .74 ± .49 min at CNO injection vs. 15.23 ± 1.52 min at vehicle injection, *n* = 8, *p* < .0001, paired *t*-test, Figure 1G). To exclude the effect of CNO on sleep–wake behavior, we tested the effect of CNO injection in the eGFP-control group, which received an AAV-GFAabc1d-eGFP injection into the PBN. Our results showed that CNO injection did not alter the sleep–wake behavior of eGFP mice compared to the vehicle injection (Supplementary Figure S3). These results illustrate that the activation of PBN astrocytes is sufficient to promote wakefulness.

### 3.2 Chemogenetic inhibition of PBN astrocytes decreases wakefulness

**FIGURE 2:**
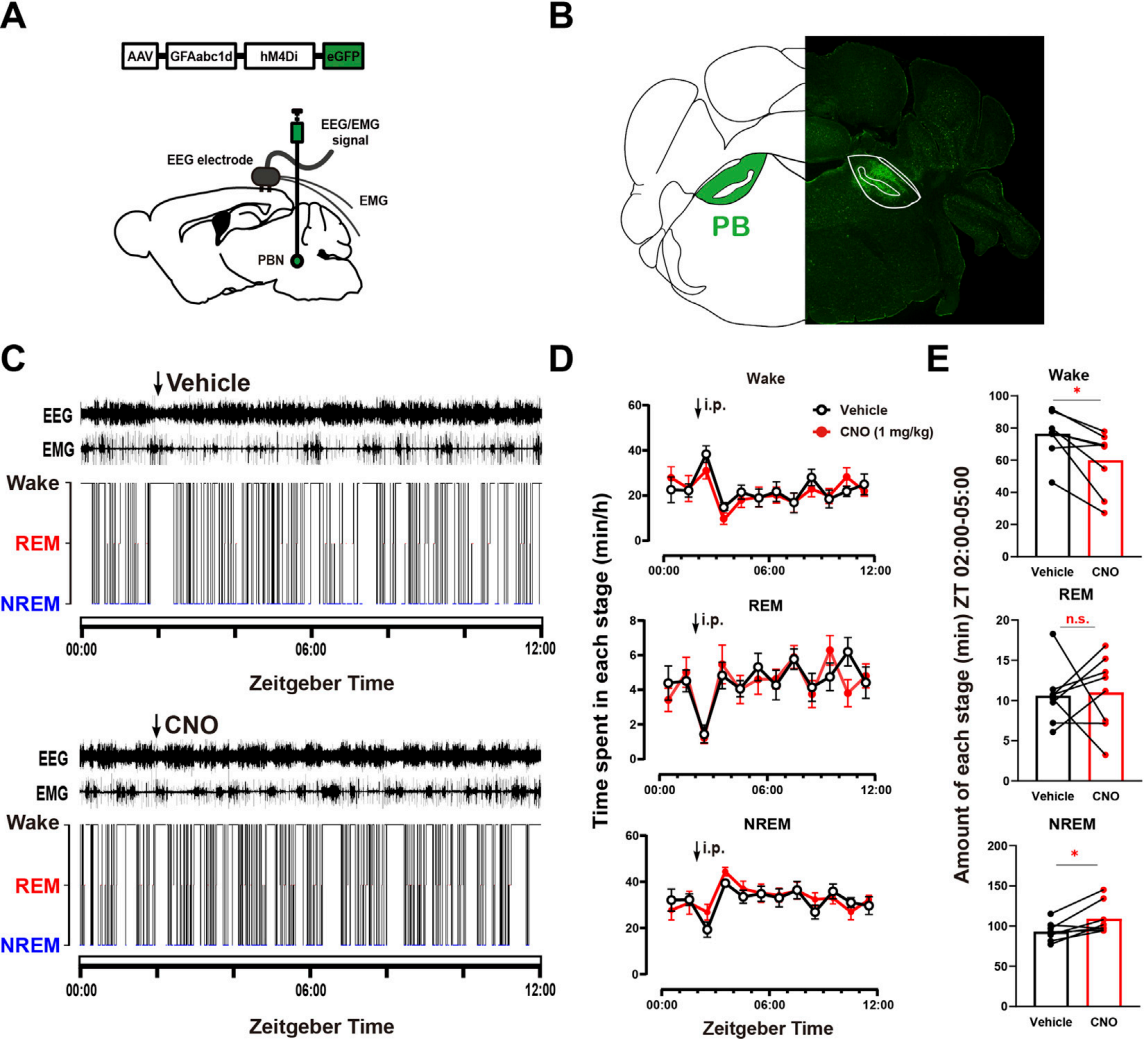
Chemogenetic inhibition of PBN astrocytes decreases total wakefulness **(A)** Schematic diagram showing the bilateral injection of AAV-GFAabc1d-hM4Di-eGFP into the PBN of mice. The EEG electrodes were screwed into the craniotomy hole, and the EMG electrodes were bilaterally placed into the trapezius muscles. **(B)** Representative fluorescent image showing the expression of AAV-GFAabc1d-hM4Di-eGFP in the PBN. **(C)** Typical examples of EEG/EMG traces and corresponding hypnograms from one hM4Di mouse which was intraperitoneally injected with vehicle (top) or CNO (1 mg/kg, bottom) successively at ZT 02:00. CNO injection was performed 24 h after vehicle injection. **(D)** Time course of wakefulness, REM sleep, and NREM sleep after intraperitoneal injection of vehicle or 1 mg/kg CNO in hM4Di mice. Two-way repeated-measures ANOVA (*n* = 8). **(E)** Total time spent in each stage during the 3-h post-injection period (ZT 02:00–05:00) in hM4Di mice. Paired *t*-test (*n* = 8). Values represent the mean ± SEM; *p < .05 shows significant differences between the vehicle and experimental groups. PBN, parabrachial nucleus; CNO, clozapine N-oxide; i.p., intraperitoneal injection.

To further illustrate the role of PBN astrocytes in sleep-wake regulation, we tested the effect of chemogeneticly inhibiting PBN astrocytes on sleep–wake behavior. To selectively inhibit PBN astrocytes, we bilaterally microinjected an inhibitory chemogenetic virus, AAV-GFAabc1d-hM4Di-eGFP, into the PBN in mice (Figure 2A). Four weeks later, the virus was expressed in the PBN (Figure 2B). Then, we injected the vehicle and CNO respectively at ZT 02:00 (Figure 2C). Our results showed that 1 mg/kg CNO injection significantly decreased the total amount of wakefulness and increased total amount of NREM sleep during the 3-h post-injection period (Wake, CNO injection 59.93 ± 6.79 min vs. Vehicle injection 76.33 ± 5.12 min, *n* = 8, *p* = .0226; NREM: CNO injection 109.0 ± 6.935 min vs. Vehicle injection 93.07 ± 4.174 min, *n* = 8, *p* = .0297; REM, CNO injection 11.01 ± 1.641 min vs. Vehicle injection 10.61 ± 1.292 min, *n* = 8, *p* = .3994, paired *t* -test, Figure 2E), although there was no significant difference in wakefulness, REM and NREM per hour (Figure 2D).

### 3.3 Chemogenetic activation of PBN astrocytes reduces isoflurane sensitivity and accelerates emergence from isoflurane anesthesia

**FIGURE 3:**
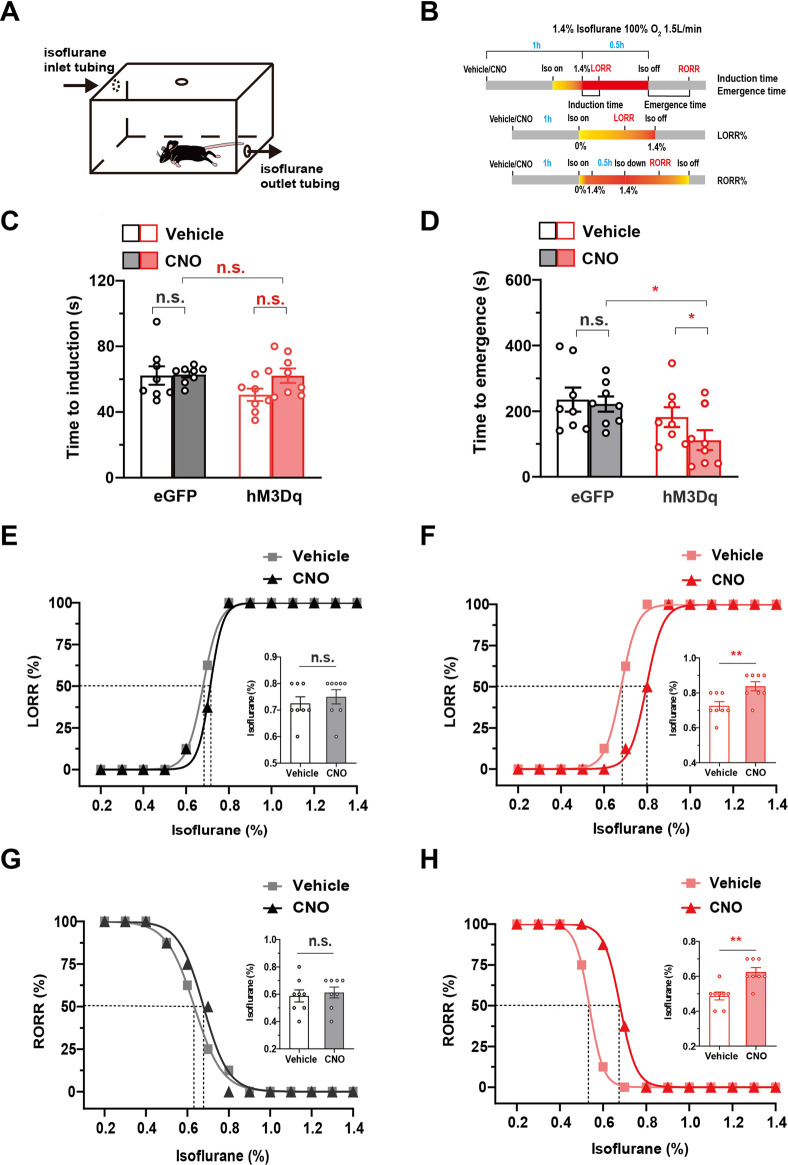
The effect of the chemogenetic activation of PBN astrocytes on the induction and emergence from isoflurane anesthesia **(A)** Schematic diagram of LORR or RORR observation during isoflurane anesthesia. **(B)**Schematic diagram of the experimental protocol to observe the effects of activation of PBN astrocytes on LORR or RORR time under 1.4% isoflurane anesthesia. **(C)** Effects of chemogenetic activation of PBN astrocytes on LORR time under 1.4% isoflurane anesthesia. Two-way repeated-measures ANOVA (*n* = 8). **(D)** Effects of chemogenetic activation of PBN astrocytes on RORR time under 1.4% isoflurane anesthesia. Two-way repeated-measures ANOVA (*n* = 8). **(E)** The dose-response curve shows the percentage of mice showing LORR as the isoflurane concentration gradually increased after vehicle or 3 mg/kg CNO injection in eGFP group. Inset: the isoflurane concentrations at which each mouse exhibited LORR are shown. Paired *t*-test (*n* = 8). **(F)** The dose-response curve shows the percentage of mice showing LORR as the isoflurane concentration gradually increased after vehicle or 3 mg/kg CNO injection in the hM3Dq group. Inset: the isoflurane concentrations at which each mouse exhibited LORR are shown. Paired *t*-test (*n* = 8). **(G)** The dose-response curve shows the percentage of mice showing RORR as the isoflurane concentration gradually decreased after vehicle or 3 mg/kg CNO injection in eGFP group. Inset: the isoflurane concentrations at which each mouse exhibited RORR are shown. Paired *t*-test (*n* = 8). **(H)** The dose-response curve shows the percentage of mice showing RORR as the isoflurane concentration gradually decreased after vehicle or 3 mg/kg CNO injection in hM3Dq group. Inset: the isoflurane concentrations at which each mouse exhibited RORR are shown. Paired *t*-test (*n* = 8). Values represent mean ± SEM; *p < .05 or **p < .01, shows significant differences between the vehicle and experimental groups. PBN, parabrachial nucleus; CNO, clozapine N-oxide; LORR, loss of righting reflex; RORR, recovery of righting reflex.

The above results reveal that PBN astrocytes are key neural substrates regulating wakefulness. Considering that many sleep–wake substrates have recently been shown to participate in general anesthesia (Herrera et al., 2016; Bao et al., 2021; Yang et al., 2021), we further investigated the role of PBN astrocytes in the modulation of isoflurane anesthesia. We first examined the effects of chemogenetic activation of PBN astrocytes on the induction and emergence of isoflurane anesthesia. LORR and RORR in rodents are considered surrogate indicators of anesthesia induction and recovery, respectively, in humans (Pal et al., 2015; Herrera et al., 2016). A schematic diagram of LORR or RORR assessment during isoflurane anesthesia is shown in Figure 3A, and the anesthesia behavior test protocol is shown in Figure 3B. Although sleep and anesthesia share a similar state of cortical inactivation (Franks and Zecharia, 2011), the levels of cortical activity are different during the period of sleep and anesthesia (Brown et al., 2010). Because of the inhibitive effect of general anesthetics on the cortex, the cortical activity is less active during anesthetic state, comparing with natural sleep state (Moody et al., 2021). Considering that arousal from the anesthesia state may require more strong stimulation of cortex, we used higher dose of CNO (3 mg/kg) in the anesthesia experiments. The results showed that 3 mg/kg CNO injection significantly decreased the time to emergence from isoflurane anesthesia (Time to emergence, 111.6 ± 30.24 s at CNO injection vs. 182.0 ± 30.12 s at vehicle injection in hM3Dq group; 221.1 ± 23.17 s at CNO injection vs. 234.4 ± 36.47 s at vehicle injection in eGFP group, *n* = 8, two-way repeated-measures ANOVA, F (1,14) = 5.511, *p* = .0341, Figure 3D), though did not significantly change the induction time of isoflurane anesthesia (Figure 3C). Then, we evaluated the effects of PBN astrocyte activation on sensitivity to isoflurane anesthetic. In the hM3Dq group, 3 mg/kg CNO injection significantly increased the EC50 of LORR from .6793% (95% CI 0.6739%–.6836%) to .7966% (95% CI 0.7864%–.8043%) (*n* = 8, *p* = .0016, Figure 3F) and increased the EC50 of RORR from .5361% (95% CI 0.5342%–.5379%) to .6788% (95% CI 0.6740%–.6841%) (*n* = 8, *p* = .0012, Figure 3H). In the eGFP-control group, CNO injection did not significantly increase the EC50 of LORR or RORR (*n* = 8, *p* > .05, Figures 3E, G). Collectively, these findings demonstrate PBN astrocyte activation reduces the isoflurane sensitivity and accelerates emergence from isoflurane anesthesia.

### 3.4 Chemogenetic activation of PBN astrocytes enhances cortical activity during isoflurane anesthesia

**FIGURE 4:**
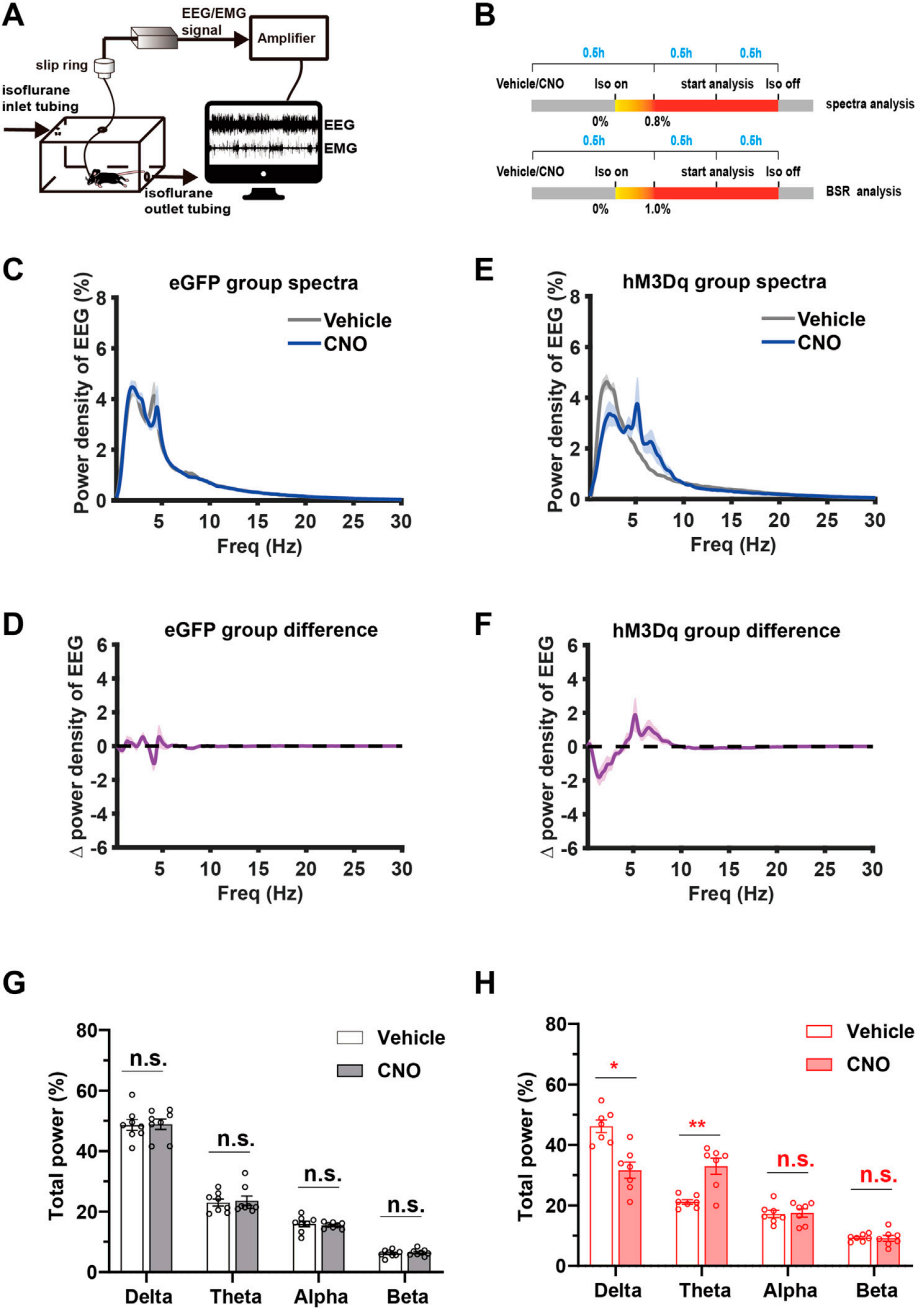
Chemogenetic activation of PBN astrocytes promotes cortical activity during isoflurane anesthesia **(A)** Schematic diagram of EEG/EMG recording during isoflurane anesthesia. **(B)** Schematic diagram of experimental protocol for observing the effect of activation of PBN astrocytes on EEG spectrum power under .8% isoflurane anesthesia and BSR under 1.0% isoflurane anesthesia.**(C)** Normalized power densities of EEG signals in the eGFP group after injection of vehicle or 3 mg/kg CNO. Shadow areas represent mean ± SEM (*n* = 8). **(D)** Differences in power densities of EEG signals in the eGFP group after injection of the vehicle and 3 mg/kg CNO. Shadow areas represent mean ± SEM (*n* = 8). **(E)**Normalized power densities of EEG signals in hM3Dq group after injection of the vehicle or 3 mg/kg CNO. Shadow areas represent mean ± SEM (*n* = 7). **(F)**Differences in power densities of EEG signals in the hM3Dq group after injection of the vehicle and 3 mg/kg CNO. Shadow areas represent mean ± SEM (*n* = 7). **(G)**Relative EEG power after vehicle or 3 mg/kg CNO injection in the eGFP group during .8% isoflurane anesthesia. Paired *t*-test (*n* = 8). **(H)** Relative EEG power after vehicle or 3 mg/kg CNO injection in the hM3Dq group during .8% isoflurane anesthesia. Paired *t*-test (*n* = 7). Values represent mean ± SEM; *p < .05 or **p < .01 shows significant differences between the vehicle and experimental groups. EEG, electroencephalogram; EMG, electromyogram; BSR, burst suppression ratio.

To investigate the effect of PBN astrocyte activation on cortical activity, we performed spectral analysis of EEG during steady-state isoflurane anesthesia. A schematic diagram of EEG/EMG recording during isoflurane anesthesia following PBN astrocyte activation is shown in Figure 4A, and the EEG/EMG recording protocol is shown in Figure 4B. Our results showed that chemogenetic activation of PBN astrocytes obviously altered the EEG power spectrum (Supplementary Figure S4). Normalized power densities of EEG signals of hM3Dq group after vehicle and CNO injection were shown in Figure 4E, and differences of normalized power densities of EEG signals between vehicle and CNO injection were shown in Figure 4F. The analysis of EEG frequency bands showed that 3 mg/kg CNO injection resulted in a marked decline in the delta power and significant increase in the theta power in the hM3Dq group. The delta band decreased from 46% ± 5% to 31% ± 7% (*n* = 7, *p* = .011, paired *t*-test), and the theta band increased from 21% ± 2% to 33% ± 7% (*n* = 7, *p* = .0064, paired *t*-test, Figure 4H). In eGFP-control mice, CNO administration did not significantly change the normalized EEG power spectrum (Figures 4C,D) or the EEG frequency bands (Figure 4G).

**FIGURE 5:**
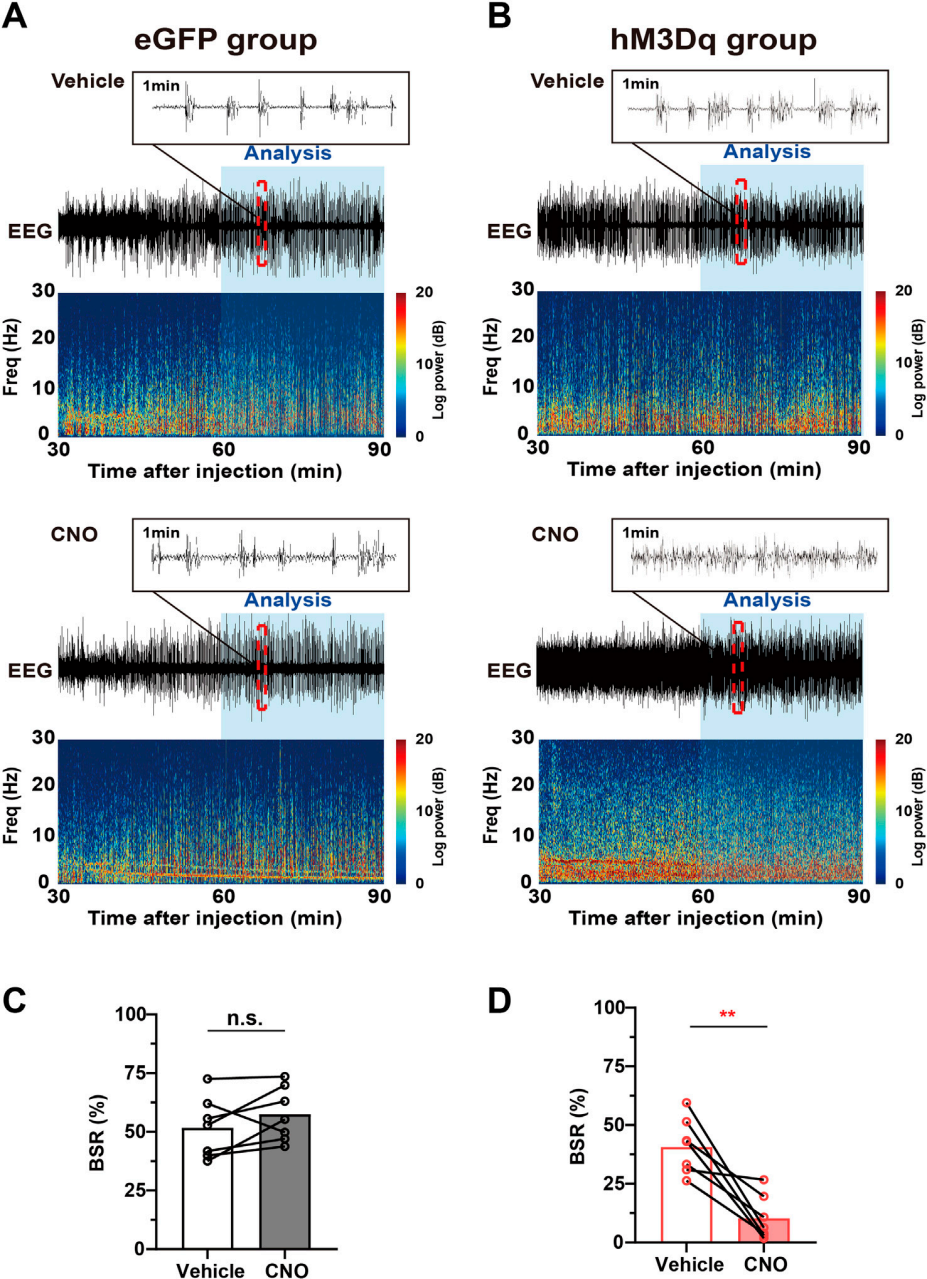
Chemogenetic activation of PBN astrocytes decreases the BSR under 1.0% isoflurane anesthesia. **(A)** Schematic diagram of representative burst-suppression oscillation of the eGFP group after vehicle (top) or CNO (3 mg/kg, bottom) injection under 1.0% isoflurane anesthesia. **(B)** Schematic diagram of representative burst-suppression oscillation of the hM3Dq group after vehicle (top) or CNO (3 mg/kg, bottom) injection under 1.0% isoflurane anesthesia. **(C)** Statistics showing the change in the BSR after vehicle and 3 mg/kg CNO injection in the eGFP group during 1.0% isoflurane anesthesia. Paired *t*-test (*n* = 7). **(D)** Statistics showing the change in the BSR after vehicle and 3 mg/kg CNO injection in the hM3Dq group during 1.0% isoflurane anesthesia. Paired *t*-test (*n* = 7). Values represent mean ± SEM; *p < .05 or **p < .01 shows significant differences between the vehicle and experimental groups. BSR, burst-suppression-ratio.

The burst-suppression mode in EEG features high-voltage bursts alternated with low-voltage suppression and is a clinically observed pattern indicating the depth of drug-induced anesthesia during maintenance. In animal experiments, the BSR is widely used to evaluate the change in cortical excitability during inhalational general anesthesia (Yin et al., 2019; Bao et al., 2021). One hour after vehicle or 3 mg/kg CNO injection, mice were exposed to 1.0% isoflurane for 30 min, the EEG/EMG signal was recorded, and the BSR was calculated. A schematic diagram of representative burst-suppression EEG of the eGFP and hM3Dq group after vehicle and CNO injections under 1.0% isoflurane anesthesia is shown in Figures 5A,B. Our results showed that CNO injection elicits a dramatic decrease in BSR in the hM3Dq group (CNO vs. vehicle, 10% ± 4% vs. 41% ± 12%, *n* = 7, *p* = .0034, paired *t*-test, Figure 5D). In eGFP mice, CNO injection did not significantly change the BSR (CNO vs. vehicle, 57.49% ± 4.37% vs. 51.74% ± 4.871%, *n* = 7, *p* > .05, paired *t*-test, Figure 5C). These findings collectively demonstrate that PBN astrocyte activation changes the EEG spectrum power and decreases the BSR, indicating a decreased depth of anesthesia and enhanced cortical activity during isoflurane anesthesia.

### 3.5 Chemogenetic inhibition of PBN astrocytes alters isoflurane anesthesia sensitivity and cortical activity

**FIGURE 6:**
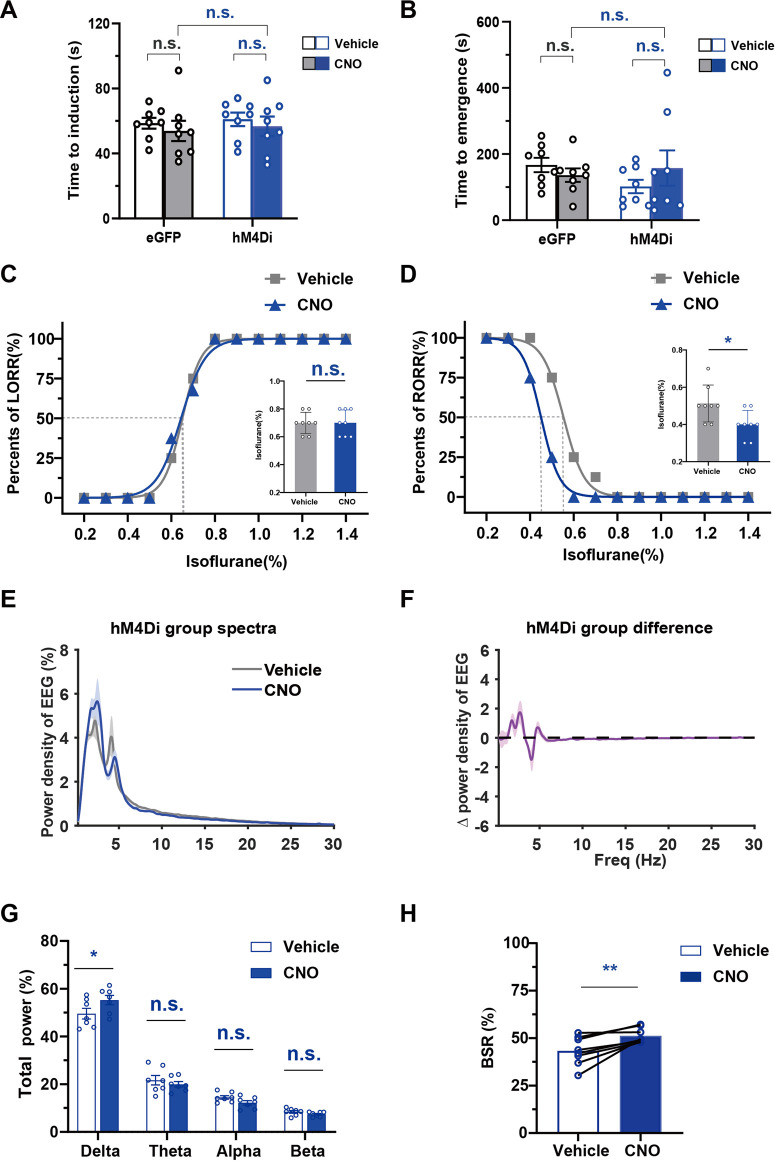
The effect of chemogenetic PBN astrocyte inhibition on the induction and emergence from isoflurane anesthesia, and the EEG under isoflurane anesthesia **(A)** Effects of chemogenetic inhibition of PBN astrocytes on the LORR time under 1.4% isoflurane anesthesia. Two-way repeated-measures ANOVA (*n* = 8). **(B)** Effects of chemogenetic inhibition of PBS astrocytes on the RORR time under 1.4% isoflurane anesthesia. Two-way repeated-measures ANOVA (*n* = 8). **(C)** The dose-response curve shows the percentage of mice showing LORR as the isoflurane concentration gradually increased after vehicle or 3 mg/kg CNO injection in hM4Di group. Inset: the isoflurane concentrations at which each mouse exhibited LORR are shown. Paired *t*-test (*n* = 8). **(D)** The dose-response curve shows the percentage of mice showing RORR as the isoflurane concentration gradually decreased after vehicle or 3 mg/kg CNO injection in hM4Di group. Inset: the isoflurane concentrations at which each mouse exhibited RORR are shown. Paired *t*-test (*n* = 8). **(E)** Normalized power densities of EEG signals in hM4Di group after injection of the vehicle or 3 mg/kg CNO. Shadow areas represent mean ± SEM (*n* = 7). **(F)**Differences in power densities of EEG signals in hM4Di group after injection of 3 mg/kg CNO and the vehicle. Shadow areas represent mean ± SEM (*n* = 7). **(G)** Relative EEG power after vehicle or 3 mg/kg CNO injection in hM4Di group during .8% isoflurane anesthesia. Paired *t*-test (*n* = 7). **(H)** Statistics showing the change in BSR after vehicle and 3 mg/kg CNO injection in hM4Di group during 1.0% isoflurane anesthesia. Paired *t*-test (*n* = 8). Values represent the mean ± SEM; *p < .05, **p < .01 shows significant differences between the vehicle and experimental groups.

To examine the effects of chemogenetic inhibition of PBN astrocytes on isoflurane anesthesia, we bilaterally injected AAV-GFAabc1d-hM4Di-eGFP into the PBN of mice. Our results showed that injection of 3 mg/kg CNO did not significantly alter the induction time of isoflurane anesthesia (CNO vs. vehicle in the hM4Di group, 56.75 ± 6.044 s vs. 61.00 ± 4.136 s; CNO vs. vehicle in eGFP group, 53.88 ± 6.229 s vs. 58.63 ± 3.359 s; *n* = 8, two-way repeated-measures ANOVA, F (1,14) = 1.523, *p* = .2375, Figure 6A) and the emergency time from isoflurane anesthesia (CNO vs. vehicle in hM4Di group, 157.8 ± 53.44 s vs. 102.0 ± 20.34 s; CNO vs. vehicle in eGFP group 136.4 ± 20.48 s vs. 167.1 ± 21.8 s; *n* = 8, two-way repeated-measures ANOVA, F (1,14) = .1736, *p* = .6833, Figure 6B). However, inhibition of PBN astrocytes resulted in a significant decrease in the EC50 of RORR (*n* = 8, *p* = .0256, Figure 6D) from .5532% (95% CI 0.5419%–.5646%) to .4500% (95% CI 0.4447%–.4553%), although it did not significantly change the EC50 of LORR (*n* = 8, *p* = .3149, Figure 6C).

We further analyzed the change in EEG spectrum power after the inhibition of PBN astrocyte (Figures 6E,F). The analysis of EEG frequency bands showed that PBN astrocytes inhibition significantly increased the delta power (Delta, 55.25% ± 1.967% at CNO injection vs. 49.55% ± 2.215% at vehicle injection, *n* = 7, *p* < .05; Theta, 19.96% ± 1.136% at CNO injection vs. 21.65% ± 1.995% at vehicle injection, *n* = 7, *p* > .05; Alpha, 12.2% ± .9129% at CNO injection vs. 14.47% ± .7673% at vehicle injection, *n* = 7, *p* > .05; Beta, 7.396% ± .3826% at CNO injection vs. 8.401% ± .5625% at vehicle injection *n* = 7, *p* > .05, paired *t*-test, Figure 6G) during .8% isoflurane anesthesia. During 1.0% isoflurane anesthesia, PBN astrocyte inhibition elicited a significant increase in the BSR (CNO vs. vehicle, 51.22% ± 1.388% vs. 43.29% ± 2.647%, *n* = 8, *p* = .0046, paired *t*-test, Figure 6H). In the eGFP group, CNO injection did not significantly change the EEG spectrum power (Supplementary Figures S5A–C) or BSR (Supplementary Figure S5D) during isoflurane anesthesia compared with the vehicle injection group. Taken together, these findings indicate that inhibition of PBN astrocytes increases isoflurane sensitivity and decreases cortical activity during isoflurane anesthesia.

## 4 Discussion

In the present study, using a chemogenetic approach, we selectively manipulated PBN astrocytes to elucidate their regulatory effects on sleep–wake behavior and isoflurane-induced general anesthesia. Our results showed that the chemogenetic activation of PBN astrocytes strongly promoted wakefulness, while inhibiting PBN astrocytes decreased wakefulness and increased sleep duration significantly. Regarding isoflurane-induced anesthesia, we found that chemogenetic activation of PBN astrocytes shortened the emergence time and decreased anesthesia sensitivity. The BSR analysis further revealed the attenuated depth of sedation after PBN astrocyte activation. Chemogenetic inhibition of PBN astrocytes increased the EEG delta power and BSR, indicating attenuated cortical activity. Our findings clearly illustrate that PBN astrocytes are involved in regulating sleep–wake behavior and isoflurane anesthesia.

Previous studies have focused on the role of CNS neurons in sleep–wake regulation, while recent studies have shown that astrocytes in the CNS also participate in regulating sleep–wake behavior. Fiber photometry results showed that the calcium signaling of cortex astrocytes fluctuated dynamically with vigilance states, which are the highest in wake states and lowest during sleep (Ingiosi et al., 2020). Decreased astrocytic calcium levels due to knockout of STIM1 reduced sleep drive after sleep deprivation in mice (Ingiosi et al., 2020). Previous results have shown that the optogenetic activation of astrocytes in the ventrolateral preoptic area, a key sleep-promoting structure, increases the active phase sleep duration in adult male rats (Kim et al., 2020). In contrast to the sleep-promoting effect of ventrolateral preoptic area astrocytes, our results showed that PBN astrocyte activation potently promotes wakefulness. The different regulatory effects of PBN and VLOP astrocytes imply the heterogeneity of CNS astrocytes in sleep–wake regulation. Recent results have shown that the calcium dynamics of astrocytes varies depending on brain regions during sleep-wake behavior (Tsunematsu et al., 2021). Transcriptional analyses showed that the heterogeneity in astrocytic gene expression exists not only among but within the brain regions (Chai et al., 2017; Zeisel et al., 2018; Batiuk et al., 2020; Bayraktar et al., 2020). The heterogeneity of CNS astrocytes may partly explain the different regulatory roles of CNS astrocytes in sleep-wake regulation.

In the current study, our results show that PBN astrocytes not only regulate sleep–wake behavior but also isoflurane-induced general anesthesia, revealing an overlapping mechanism underlying wakefulness and anesthetic emergence. In addition to PBN, many arousal-related neural substrates have been shown to be involved in regulating reanimation from general anesthesia. For example, optogenetic stimulation of VTA dopaminergic neurons generates long-time wakefulness in mice and restores the righting reflex after isoflurane-induced general anesthesia (Taylor et al., 2016). Activation of the glutamatergic neurons in the lateral hypothalamus induces instant wakefulness from sleep, lengthens the general anesthesia induction time and accelerates emergence from general anesthesia (Wang et al., 2021; Zhao et al., 2021). Additionally, the activation of noradrenergic neurons in the locus coeruleus induces instant arousal and markedly facilitates behavioral emergence from general anesthesia (Vazey and Aston-Jones, 2014). The evidence from current and prior studies suggests that there are some overlapping neural substrates modulating sleep–wake behavior and general anesthesia. Further into investigation of the role of arousal-promoting neural substrates, with both neurons and astrocytes included, in the regulation of reanimation from general anesthesia may help elucidate the exact mechanisms of general anesthesia.

In this study, chemogenetic activation of PBN astrocytes obviously suppressed the BSR and decreased cortical slow oscillation in mice. These results indicate that PBN astrocytes affect the depth of anesthesia and enhance cortical excitability. Previous results have shown that activation of PBN glutamatergic neurons increases c-Fos expression in the cerebral cortex, especially in the prefrontal and motor cortices, during sevoflurane anesthesia (Wang et al., 2019). Further immunohistochemical staining results showed that PBN glutamate neuron activation strongly excites subcortical key arousal nuclei, such as in the BF and LH. Neuroanatomical results showed that PBN projections directly project to the BF and LH (Qiu et al., 2016). Viral-mediated retrograde activation of PBN-BF and PBN-LH ascending circuit pathways strongly promotes cortical arousal and behavioral wakefulness in mice (Qiu et al., 2016). Given that the BF and LH directly project to the cortex and regulate cortical activity (Anaclet et al., 2015; Bonnavion et al., 2016; Chen et al., 2016; Arrigoni et al., 2018), it is possible that the BF and LH may participate in the modulation of BSR and cortical excitability by PBN astrocytes.

## Data Availability

The original contributions presented in the study are included in the article/Supplementary Material, further inquiries can be directed to the corresponding authors.
