# Supplementary material for: Parabrachial nucleus astrocytes regulate wakefulness and isoflurane anesthesia in mice
Source: Front Pharmacol. 2023 Jan 13;13:991238. doi: 10.3389/fphar.2022.991238 (PMC9880442; doi:10.3389/fphar.2022.991238)
Supplement: Supplementary file 1 [file DataSheet1.docx]

**Tittle:** Parabrachial nucleus astrocytes regulate wakefulness and isoflurane anesthesia in mice

**Abbreviated title:** PBN astrocytes regulate wakefulness and GA

**Authors:**

Pei-Chang Liu^1^, Wei Yao^2^, Xing-Yu Chen^2^, Wei-Kun Su^3^, Ze-Hong Zheng^4^, Xiong-Bin Yan^4^, Ya-Ling Deng^4^, Kai-Ge Shi^4^, Xin Liu^4^, Yu-Wei Gao^4^, Tian-Tian Lin^4^, Yun-Xi Zhu^4^, Ying-Xuan Lin^4^, Zhong-Hua Zhu^4^, Ping Cai^2^*, Liang-Cheng Zhang^1^*, and Li Chen^3^*

**Affiliations:**

^1^Department of Anesthesiology, Fujian Medical University Union Hospital, Fuzhou 350001, Fujian, China;

^2^Fujian Province Key Laboratory of Environment and Health, School of Public Health, Fujian Medical University, Fuzhou 350108, Fujian, China;

^3^Department of Pharmacology, School of Pharmacy, Fujian Medical University, Fuzhou 350108, Fujian, China;

^4^ School of Basic Medical Sciences, Fujian Medical University, Fuzhou 350108, Fujian, China;

Pei-Chang Liu, Wei Yao, Xing-Yu Chen and Wei-Kun Su contributed equally to this work.

***Corresponding authors:** lichen01005@163.com (Li Chen), [drzhang2017@sina.com](mailto:drzhang2017@sina.com) (Liang-Cheng Zhang) or caipingfjmu@163.com (Ping Cai)

**Supplementary Material**


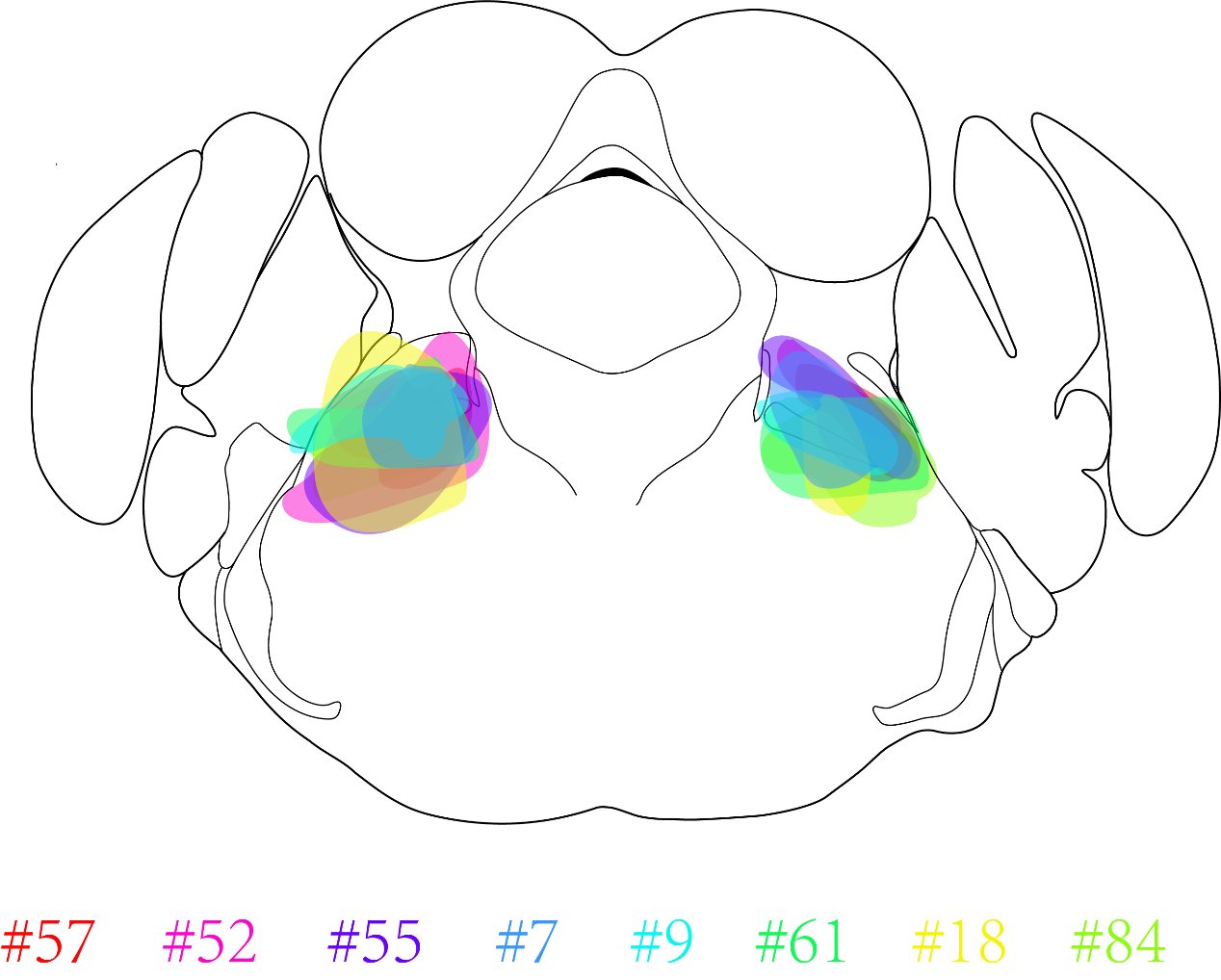


**Supplementary Figure** **1. Drawings of superimposed hM3Dq-eGFP expression in the PBN.** The AAV-gfaABC1D-hM3Dq-eGFP was injected into the PBN of mice, and the expression extent of hM3Dq-eGFP was checked after behavioral testing (n=8, indicated with different colors).

**
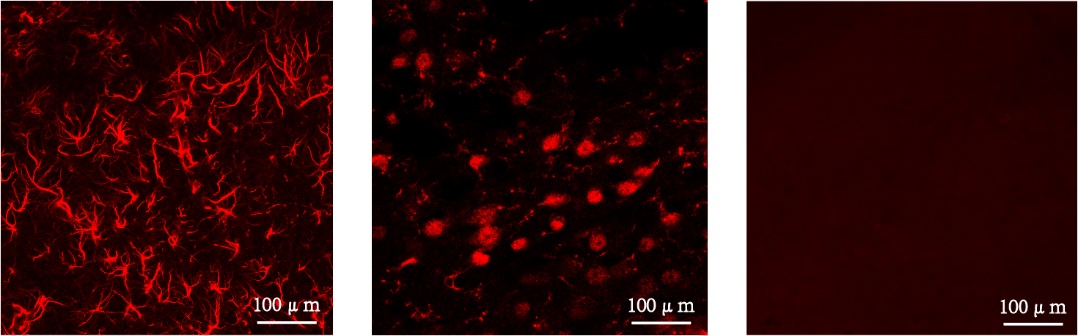
**

**Supplementary Figure** **2.** **Donkey-anti-mouse-Alexa Fluor 594 (secondary antibodies) does not result in false positive immunolabeling.** Schematic diagram of staining with mouse-anti-GFAP antibody and donkey-anti-mouse-Alexa Fluor 594 (secondary antibodies) (left), mouse-anti-NeuN antibody and donkey-anti-mouse-Alexa Fluor 594 (secondary antibodies) (middle), and donkey-anti-mouse-Alexa Fluor 594 [alone](https://dict.youdao.com/w/individual/#keyfrom=E2Ctranslation) (secondary antibodies) (right).


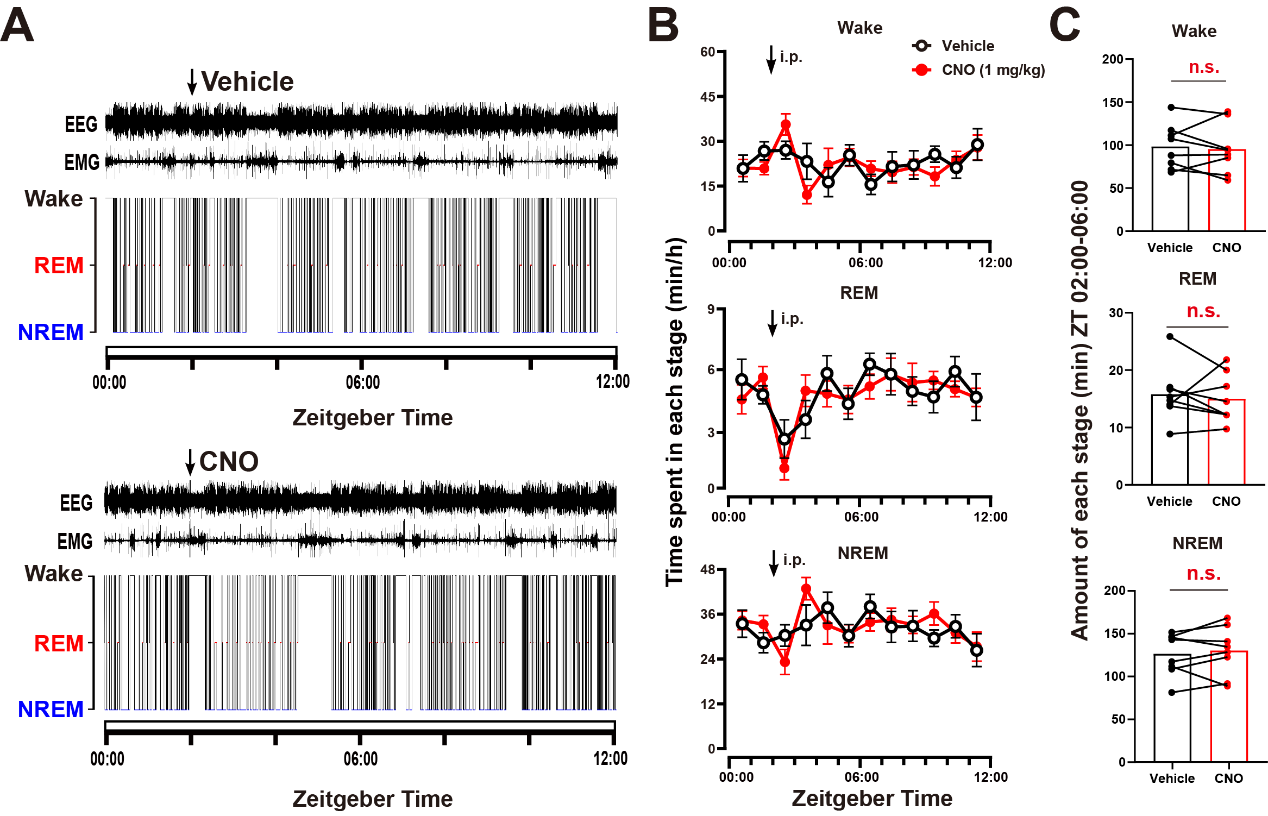


**Supplementary Figure** **3. CNO injection did not change sleep-wake behavior in eGFP-control mice.**

(A) Typical examples of EEG/EMG traces and corresponding hypnograms from one eGFP-control mouse which was intraperitoneally injected with vehicle (top) or CNO (1 mg/kg, bottom) successively at ZT 02:00. CNO injection was performed 24 h after vehicle injection.

(B) Time course of wakefulness, REM sleep, and NREM sleep after intraperitoneal injection of vehicle and CNO in eGFP-control mice. Two-way repeated-measures ANOVA (n = 8).

(C) Total time spent in each stage during the 4-hr post-injection period (ZT 02:00-06:00) in eGFP-control mice. Paired *t*-test (n = 8).

Values represent mean ± SEM, *P < 0.05, shows significantly different between vehicle group and experimental group. PBN, parabrachial nucleus; CNO, clozapine N-oxide.


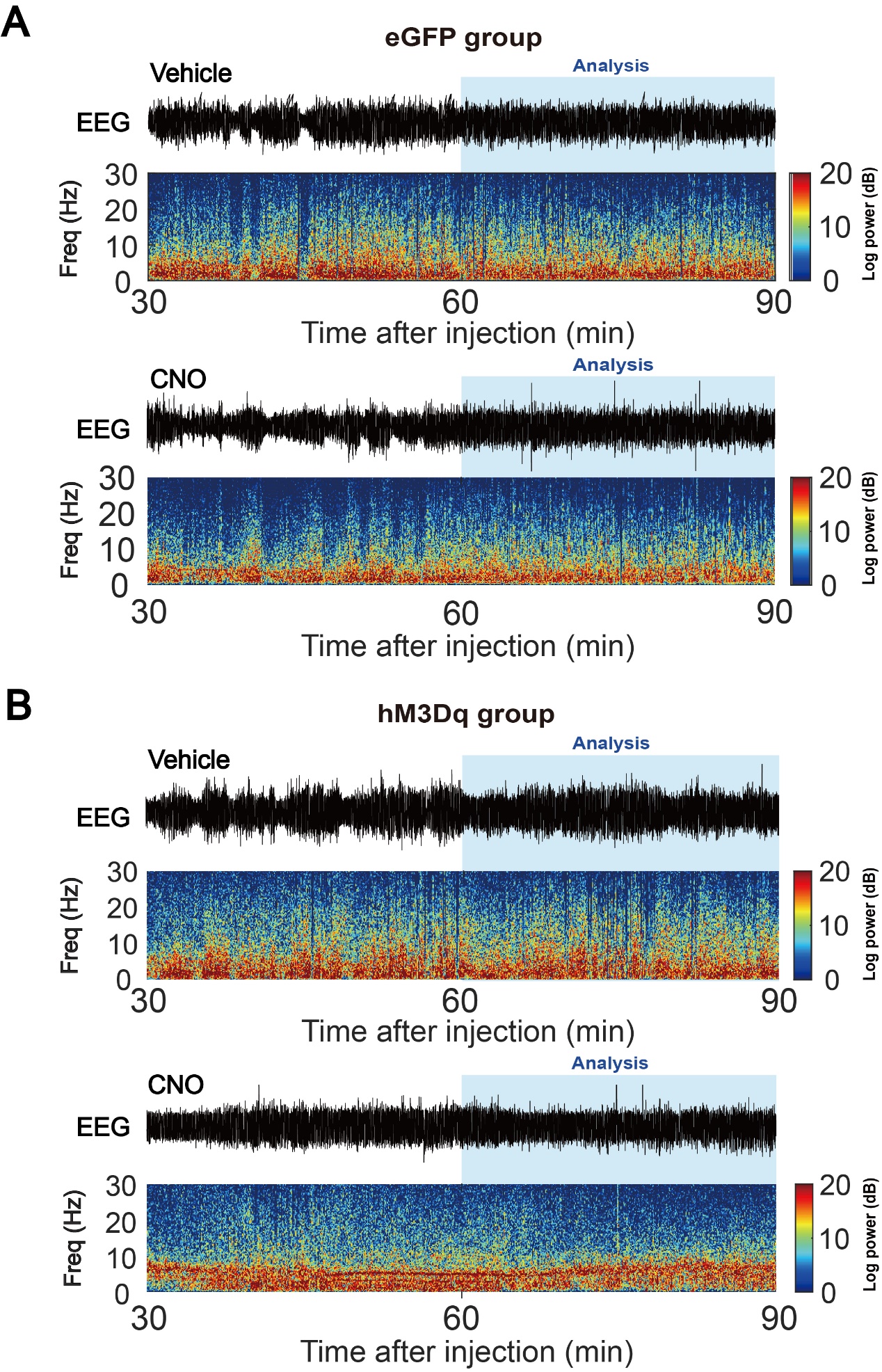


**Supplementary Figure 4. Typical diagram of** **EEG spectrum power in eGFP and hM3Dq mice anesthetized with 0.8% isoflurane.**

(A) Typical diagram of EEG spectrum power in the eGFP group after vehicle (top) or CNO (3 mg/kg, bottom) injection under 0.8% isoflurane anesthesia.

(B) Typical diagram of EEG spectrum power in the hM3Dq group after vehicle (top) or CNO (3 mg/kg, bottom) injection under 0.8% isoflurane anesthesia.


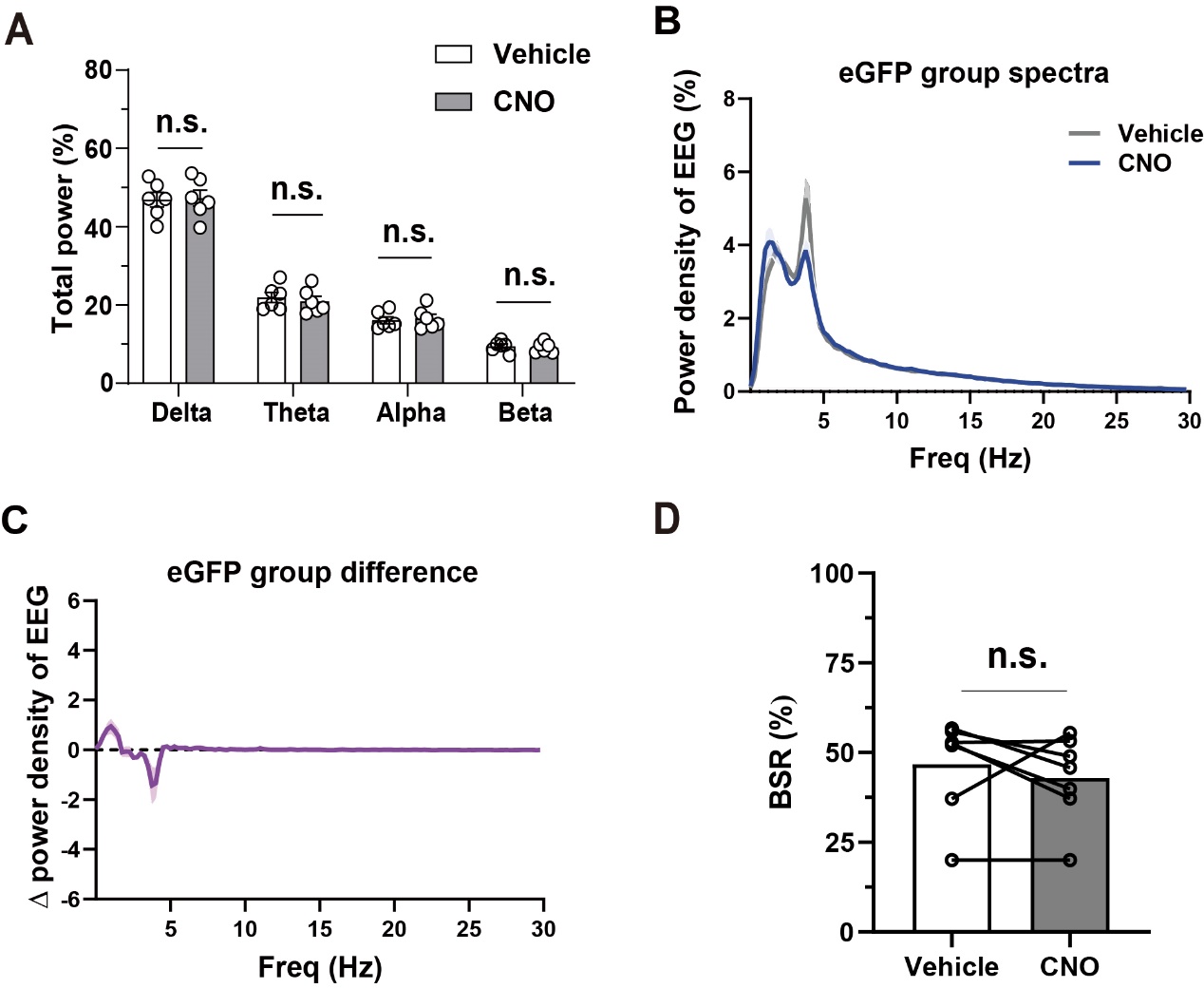


**Supplementary Figure** **5. CNO injection did not change EEG power and relative BSR in eGFP-control mice.**

(A) Relative EEG power after vehicle or CNO injection in eGFP group during 0.8% isoflurane anesthesia. Paired *t*-test (n = 6).

(B) Normalized power densities of EEG signals in eGFP group after injection of vehicle or CNO. Shadow areas represent mean ± SEM (n = 6).

(C) Differences in power densities of EEG signals in eGFP group between after injection of CNO and vehicle. Shadow areas represent mean ± SEM (n = 6).

(D) Relative BSR after vehicle or CNO injection in eGFP group during 1.0% isoflurane anesthesia. Paired *t*-test (n = 7).
